# Comparison of global DNA methylation analysis by whole genome bisulfite sequencing and the Infinium Mouse Methylation BeadChip using fresh and fresh-frozen mouse epidermis

**DOI:** 10.1080/15592294.2022.2144574

**Published:** 2022-11-14

**Authors:** Olivia Strunge Meyer, Mikkel Meyer Andersen, Claus Børsting, Niels Morling, Hans Christian Wulf, Peter Alshede Philipsen, Catharina Margrethe Lerche, Jeppe Dyrberg Andersen

**Affiliations:** a Section of Forensic Genetics, Department of Forensic Medicine, Faculty of Health and Medical Sciences, University of Copenhagen, 2100 Copenhagen, Denmark; b Department of Mathematical Sciences, Aalborg University, 9220 Aalborg, Denmark; c Department of Dermatology, Copenhagen University Hospital - Bispebjerg and Frederiksberg, 2400 Copenhagen, Denmark; d Department of Pharmacy, University of Copenhagen, 2100 Copenhagen, Denmark

**Keywords:** Whole genome bisulphite sequencing, Infinium Mouse Methylation BeadChip, DNA methylation, epidermis, hairless mice, CpG methylation

## Abstract

Until recently, studying the murine methylome was restricted to sequencing-based methods. In this study we compared the global DNA methylation levels of hairless mouse epidermis using the recently released Infinium Mouse Methylation BeadChip from Illumina and whole genome bisulphite sequencing (WGBS). We also studied the effect of sample storage conditions by using fresh and fresh-frozen epidermis. The DNA methylation levels of 123,851 CpG sites covered by both the BeadChip and WGBS were compared. DNA methylation levels obtained with WGBS and the BeadChip were strongly correlated (Pearson correlation r = 0.984). We applied a threshold of 15 reads for the WGBS methylation analysis. Even at a threshold of 10 reads, we observed no substantial difference in DNA methylation levels compared with that obtained with the BeadChip. The DNA methylation levels from the fresh and the fresh-frozen samples were strongly correlated when analysed with both the BeadChip (r = 0.999) and WGBS (r = 0.994). We conclude that the two methods of analysis generally work equally well for studies of DNA methylation of mouse epidermis and find that fresh and fresh-frozen epidermis can generally be used equally well. The choice of method will depend on the specific study’s aims and the available resources in the laboratory.

## Introduction

DNA methylation is a common epigenetic modification regulating gene expression [19]. Most often, a methyl group (CH_3_) is added to a cytosine nucleotide followed by a guanine nucleotide (CpG sites). Reliable techniques for analysis and quantification of DNA methylation are essential in many genetic and epigenetic research fields. The most common methods require sodium bisulphite (NaHSO_3_) treatment of DNA as the first step [3]. Bisulphite treatment distinguishes methylated and unmethylated cytosines by chemically converting all unmethylated cytosines into uracils, while methylated cytosines are unaffected [7]. In a subsequent PCR, uracils are converted to thymines. Thus, the methylation level of the cytosine can be determined with SNP typing methods [7]. It is well known that bisulphite treatment leads to loss of DNA and DNA degradation [3,4]. Thus, sensitive methods for the subsequent DNA methylation analysis are required.

DNA methylation has been widely studied in humans with microarrays enabling simultaneous analysis of hundreds of thousands of CpG sites [2,3]. The Illumina Infinium Mouse Methylation BeadChip was recently launched, enabling analysis of more than 285,000 CpG sites in the murine genome [27]. With the BeadChip method, bisulphite-treated DNA fragments bind to hybridization probes complementary to fragments with preselected CpG sites. The probes hybridize directly upstream of the CpG site to enable single-base extension of the probe with a fluorescent di-deoxy nucleotide. The extended base emits a fluorescent signal (green for C/G and red for T/A), and the methylation level (reported as a β-value) is estimated from the fluorescence [2]. Whole genome bisulphite sequencing (WGBS) theoretically enables the analysis of all CpG sites in the genome. The methylation level of a CpG site can be calculated as, e.g., the C/T ratio or the C/(C + T) proportion based on the read counts [3].

The methylation status of specific CpG sites has been used as biomarkers for, e.g., tissue identification and biological age in humans [9,12,21]. Moreover, DNA methylation patterns have been shown to be associated with many diseases such as cancers [11,17] and heart diseases [1,14]. Various human DNA methylation analysis methods have been carefully evaluated and compared [5,10,22]. Until recently, commercially available methods for studying the murine methylome were restricted to sequencing-based methods. In this study, we compared the global DNA methylation levels of mouse epidermis using WGBS and the new Infinium Mouse Methylation BeadChip kit (Illumina) and evaluated the concordance between the two methods. The analysis was carried out using paired fresh and fresh-frozen epidermis to investigate possible sample storage effects on DNA methylation. Previous studies have focused on comparing DNA methylation analyses using fresh-frozen and formalin-fixed and paraffin-embedded (FFPE) tissue samples [15,20]. In humans and mice, strong concordance has been reported between DNA methylation levels from FFPE and fresh-frozen tissue samples [6,8,15,20,27]. Yet, using fresh-frozen tissue is the golden standard for DNA methylation analysis. Here, we assessed the correlation between DNA methylation levels obtained from fresh-frozen and fresh epidermis from the same mouse.

## Materials and Methods

### Mouse samples

Six immunocompetent female C3.Cg-*HR^hr^*/Tac hairless mice (Taconic) ages 17–23 weeks were included in the study. The skin was removed from the back of the mice immediately after the mice were euthanized in a CO_2_-atmosphere. The skin was placed in sodium chloride solution (58.44 g/L) at room temperature for 24 hours. The epidermis was gently removed from the dermis using a tweezer. We analysed a fresh and a fresh-frozen sample from each mouse. For fresh samples, DNA was extracted directly from the fresh epidermis. For fresh-frozen samples, the epidermis was frozen at −80°C for 4–6 weeks before DNA extraction. Each sample was analysed with both the Infinium Mouse Methylation BeadChip and WGBS resulting in a total of 24 analysed samples. The study was approved by the Danish Animal Experiments Inspectorate (2019–15-0201-00131) and carried out in accordance with the guidelines described in the EU Directive 2010/63/EU for animal experiments.

### DNA extraction

DNA was extracted using the DNeasy Blood and Tissue kit from Qiagen. A small piece of the fresh or thawed fresh-frozen epidermis was added to an Eppendorf tube with 180 µL ATL buffer, 40 µL Proteinase K, and a 5 mm stainless steel bead (Qiagen). The epidermis was disrupted in a TissueLyser II (Qiagen) for 2 min with standard settings (20.0 Hz). Subsequently, the DNA was extracted following steps 3–7 in the Spin-Column Protocol for Purification of Total DNA from Animal Tissues (Qiagen, July 2020). DNA was quantified using the HS dsDNA Assay kit for fluorometric quantification on a Qubit 4 Fluorometer (Thermo Fisher Scientific). The same DNA extract was used for both WGBS and BeadChip analyses.

### Infinium Mouse Methylation BeadChip

DNA methylation was analysed with the Infinium Mouse Methylation BeadChip from Illumina, following the manufacturer’s recommendations. A total of 450–500 ng genomic DNA was used for bisulphite treatment using the EZ DNA Methylation™ Kit (Zymo Research). The BeadChips were scanned using the iScan system (Illumina). Analysis was carried out in R version 4.1.2 [23] with the R-package Sensible Step-wise Analysis of DNA Methylation BeadChips (*SeSAMe*) version 1.14.2. We used default parameters and normalized the data with the *noob, pOOBAH*, and *dyeBiasCorrTypeINorm* commands [28]. β-values were retrieved from the normalized dataset using *qualityMask()* and *getBetas()* commands [28].

### Whole genome bisulphite sequencing

DNA methylation was also analysed with WGBS. For each sample, 100 ng genomic DNA was fragmented to 350 bp with the Covaris S220 Focused-ultrasonicator (Covaris). Subsequently, the DNA was bisulphite treated using the EZ DNA Methylation-Gold™ Kit (Zymo Research). WGBS libraries were prepared using the Accel-NGS® Methyl-Seq DNA Library Kit (Swift Biosciences) following the recommended instructions and five PCR cycles for the final library amplification. The WGBS libraries were sequenced using the NovaSeq platform (Illumina) and an S4 flowcell with 1% PhiX spike-in (Illumina). For five of the 12 samples, we made new WGBS libraries and sequenced the samples on an S2 flowcell with 1% PhiX spike-in due to poor quality of the original run. All samples were spiked with 0.25% unmethylated lambda phage DNA (Promega) before fragmentation. The unmethylated lambda phage DNA served as bisulphite conversion control. Base-calling was performed by analysing the raw sequencing output, bcl-files, with *bcl2fastq2* ver. 2.20 (Illumina). Adapter trimming was performed with *AdapterRemoval* ver. 2.3.2 (parameters: – qualitybase 33, – minquality 30, – minlength 30, – trimqualities, and – trimns). Since an adaptase tail was added during the library preparation, 15 bases from the end of Read1 (3’ end) and 15 bases from the start of Read2 (5’ end) were removed using the – trim3p and – trim5p commands. Reads were aligned to the mm10 (GRCm38.p6) genome, and duplicates were removed using *Bismark* version 0.22.4 with default parameters [16]. Methylation was called using *the bismark_methylation_extractor* with default parameters. All CpG sites with ≥ 1 read were called. For comparisons of BeadChip and WGBS DNA methylation, CpG sites with read counts ≥ 15 reads were accepted.

### Comparisons

The DNA methylation levels of fresh and fresh-frozen epidermis were compared for each of the six mice. DNA methylation levels obtained with the BeadChip and WGBS were compared for each of the 12 samples. For BeadChip and WGBS comparisons, the β-values were multiplied by 100 to match the percentage methylation output from *Bismark* (WGBS analysis). The genomic locations of each CpG site covered by the BeadChip were extracted from the Infinium Mouse Methylation Manifest File [13] and matched with CpG sites sequenced with WGBS while taking strand specificity into account. CpG sites were annotated to a CpG island, a CpG island shore, a CpG island shelve, or open sea (other locations in the genome) based on the GENCODEvM25 gene annotation file from *SeSAMe* [27]. All comparisons were carried out in R version 4.1.2 [23]. All correlations were assessed with Pearson’s correlation coefficient (r) using the base R *cor.test()* command. Scatter plots, boxplots, and density plots were made with the *geom_point(), geom_boxplot()*, and *geom_density_ridges()* functions in ggplot2 [26] version 3.3.5, respectively. PCA plots were made with the *autoplot(pca)* function of ggplot2.

## Results

### Descriptive results of BeadChip and WGBS methylation data

#### Infinium Mouse Methylation BeadChip

We analysed 296,070 unique probes with the Mouse Methylation BeadChip kit of which 284,862 were unique CpG sites with unique cg-numbers. The BeadChip kit also contains probes targeting SNPs, CpH sites (non-CpG sites), as well as various control probes. We normalized the BeadChip data with *SeSAMe* using default parameters. A total of 269,529 probes were shared among all 12 samples after quality filtering. Of these, 264,961 were CpG sites.

#### Whole genome bisulphite sequencing

With WGBS, the average number of reads obtained per sample was 1.28 B, of which 0.695 B (54%) were non-duplicates and uniquely aligned to the mm10 reference genome (Figure S1). We identified a total of 38,641,965 CpG sites shared among all 12 samples. This included CpG sites sequenced on both the ‘+’ and the ‘-’ strand. We accepted CpG sites with a read count of at least 15, leaving 18,067,206 CpG sites for the methylation analysis. A total of 123,851 CpG sites were covered by both the BeadChip and WGBS and passed the filtering criteria of both methods. Thus, the data used in the subsequent comparisons comprised 123,851 CpG sites. The mean number of reads per CpG site was 32. The median, 25^th^, and 75^th^ quantiles were 29, 23, and 36 reads, respectively. The bisulphite conversion control (0.25% unmethylated lambda phage DNA) showed less than 0.5% total CpG methylation in all samples.

### Comparison of fresh and fresh-frozen samples

**Figure 1.**
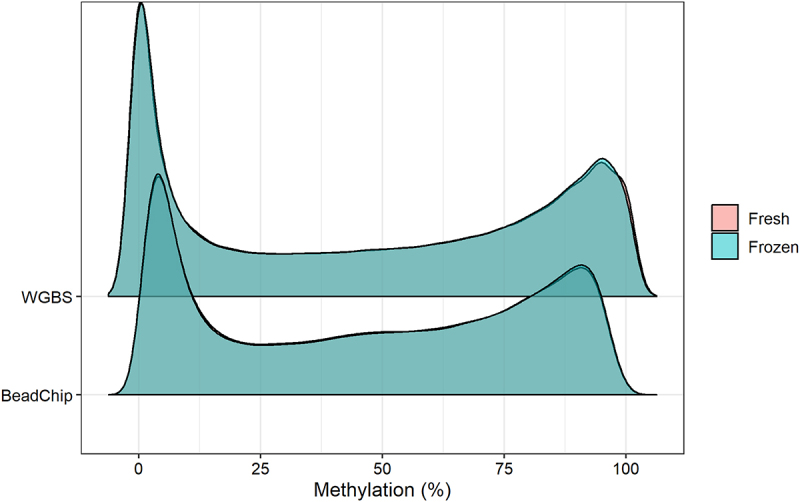
Plot of the estimated density of DNA methylation levels. The DNA methylation levels of 123,851 CpG sites was analysed from fresh and fresh-frozen epidermis of six mice with WGBS (top) and BeadChip (bottom).

We obtained 56–304 ng/µL DNA from fresh epidermis and 104–118 ng/µL DNA from fresh-frozen epidermis. We observed similar DNA methylation patterns of the fresh and the fresh-frozen samples with both analysis methods (Figure 1, Table 1). We observed a high number of CpG sites with methylation values in the two extremes, high (> 90%) and low (< 10%) DNA methylation (Figure 1).

**Table 1.** Summary of DNA methylation levels (%) of 123,851 CpG sites obtained with the BeadChip and WGBS from fresh and fresh-frozen epidermis of six mice.

	BeadChip			WGBS		
	All	Fresh	Fresh-frozen	All	Fresh	Fresh-frozen
Minimum	0.36	0.37	0.35	0.00	0.00	0.00
25^th^ quantile	12.37	12.48	12.27	7.02	7.14	6.98
Median	49.69	49.47	49.92	50.00	50.00	50.00
Mean	47.52	47.42	47.62	47.72	47.72	47.71
75^th^ quantile	78.96	78.74	79.19	84.21	84.21	84.21
Maximum	99.56	99.54	99.56	100.00	100.00	100.00

**Figure 2.**
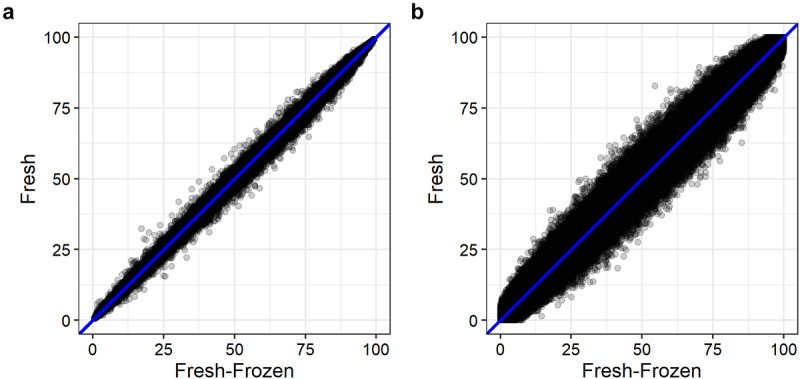
Mean DNA methylation levels of 123,851 CpG sites between fresh and fresh-frozen epidermis of six mice analysed with (a) BeadChip and (b) WGBS.

Pearson’s correlation coefficient (r) for correlation between the mean DNA methylation of fresh and fresh-frozen epidermis of six mice were r = 0.999 (r^2^ = 0.999) and r = 0.994 (r^2^ = 0.987) for samples analysed with the BeadChip and WGBS, respectively (Figure 2). We also observed strong correlations between fresh and fresh-frozen DNA methylation levels for each mouse analysed with both methods (Figure S2).

### Comparison of BeadChip and WGBS

Overall, the DNA methylation levels obtained with the BeadChip and WGBS methods were very similar (Table 1). The range of DNA methylation was wider with WGBS than with the BeadChip. The WGBS had lower 25^th^ quantile values and higher 75^th^ quantile values than the BeadChip (Table 1). The minimum and maximum methylation levels obtained with the BeadChip were never 0% and 100% methylation, as was observed for some CpG sites analysed with WGBS.

**Figure 3.**
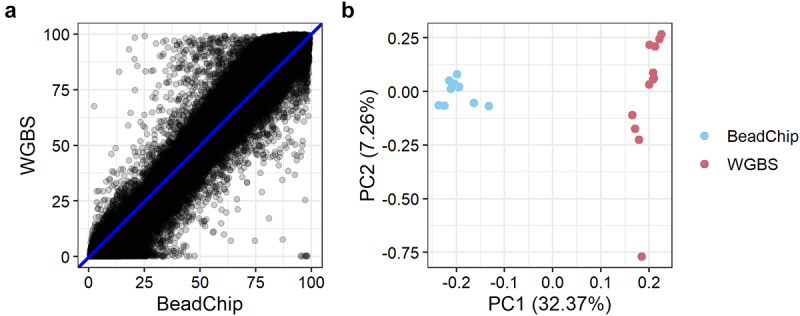
DNA methylation levels of 123,851 CpG sites analysed with BeadChip and WGBS. **a**: Mean methylation levels of 123,851 CpG sites across 12 samples analysed with BeadChip and WGBS. **b**: PCA plot of methylation levels of 12 samples analysed with BeadChip and WGBS.

Pearson’s correlation between the mean WGBS and BeadChip DNA methylation per CpG site of all 12 samples was r = 0.984 (r^2^ = 0.969) (Figure 3a). We also assessed the methylation levels obtained with WGBS and the BeadChip the with a PCA plot that showed two distinct clusters representing WGBS and BeadChip data (Figure 3b). The first principal component (PC1) explained 32.37% of the variation. The second principal component (PC2) explained 7.26% of the variation.

**Figure 4.**
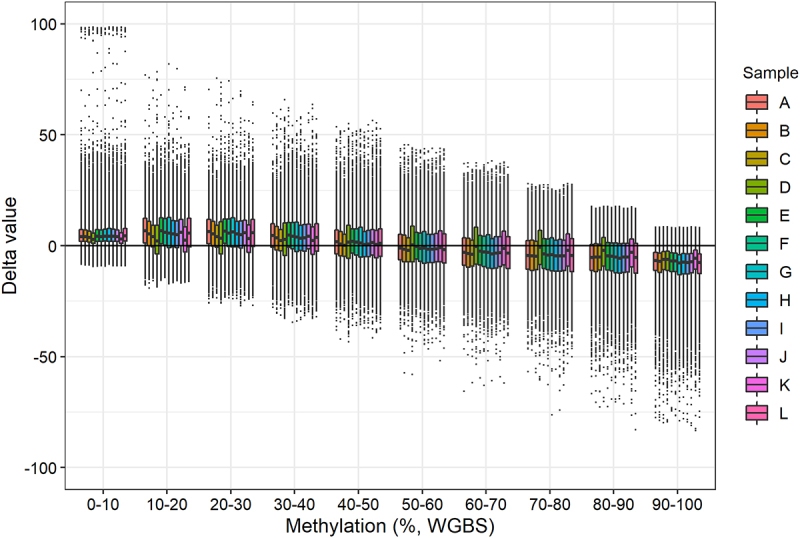
Delta values (methylation level obtained with BeadChip minus methylation level obtained with WGBS) for each sample grouped in ten WGBS methylation intervals.

We calculated delta values (methylation level obtained with BeadChip minus methylation level obtained with WGBS) for each sample at each CpG site. We then divided the data into intervals of 10% methylation (e.g., 0–10%, 10–20%, etc.) based on the WGBS methylation values (Figure 4). The mean delta values were above zero when the WGBS methylation value was 0–50% and below zero when the WGBS methylation value was 50–100%. Hence, the BeadChip DNA methylation is generally overestimated when the DNA methylation is 0–50% and underestimated when the DNA methylation is 50–100% compared with the WGBS results (Figure 4).

#### Coverage

**Figure 5.**
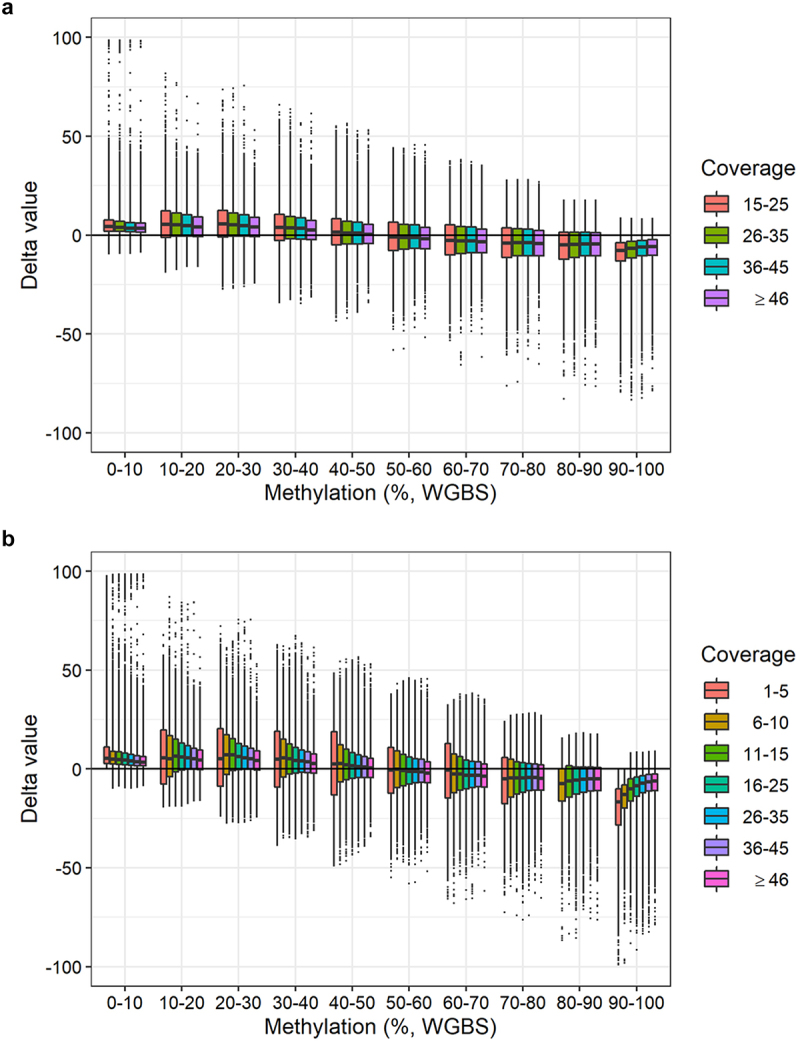
Delta values of DNA methylation levels per WGBS methylation interval stratified by WGBS coverage. **a**: Delta values (methylation level obtained with BeadChip minus methylation level obtained with WGBS) of 123,851 CpG sites per WGBS methylation interval divided into four WGBS coverage bins: 15–25, 26–35, 36–45, and ≥ 46 reads. **b**: Delta values (methylation value obtained with BeadChip minus methylation value obtained with WGBS) of 242,219 CpG sites per WGBS methylation interval divided into seven WGBS coverage bins: 1–5, 6–10, 11–15, 16–25, 26–35, 36–45, and ≥ 46 reads.

We assessed if the WGBS coverage influenced the correlation of DNA methylation levels obtained with BeadChip and WGBS. We used delta values and intervals of 10% methylation as previously described (Figure 5a). Instead of assessing each sample individually, we divided the data into four bins defined by the WGBS coverage: 15–25, 26–35, 36–45, and ≥ 46 reads (Figure 5a). We observed no substantial effect of the coverage on the delta values. We also analysed BeadChip CpG sites with WGBS coverage ≥ 1 read. This resulted in the analysis of 242,219 CpG sites. Again, we used delta values and intervals of 10% methylation, but divided the data into seven bins defined by the WGBS coverage: 1–5, 6–10, 11–15, 16–25, 26–35, 36–45, and ≥ 46 reads (Figure 5b). We observed a slightly wider spread of delta values for CpG sites covered by 1–10 reads (bins 1–5 and 5–10) (Figure 5b). This was also the case for CpG sites covered by 11–15 reads when the methylation was 10–80%. However, overall, the delta values within each coverage bin were similar (Figure 5b). This showed that a threshold of 15 reads was sufficient for this study.

#### CpG locations

**Figure 6.**
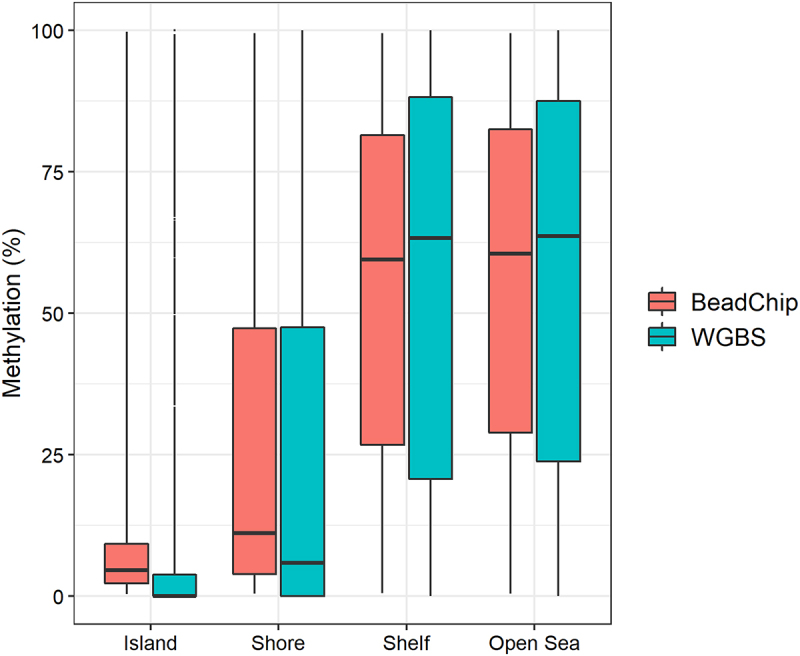
DNA methylation levels of 123,851 CpG sites based on the CpG site location in either CpG Islands (Island), CpG island shores (Shore), CpG island shelves (Shelf), or other genomic locations (Open Sea) analysed with BeadChip and WGBS.

We classified the 123,851 CpG sites into CpG islands (n = 13,032), CpG island shores (n = 14,103), CpG island shelves (n = 5,270), or open sea regions (n = 91,446) based on the annotation file provided by *SeSAMe* [27]. For CpG sites located in CpG islands and CpG island shores, the median BeadChip methylation levels were slightly higher than the median WGBS methylation levels (Figure 6). The opposite trend was observed for CpG sites located in CpG island shelves and open sea regions (Figure 6). We found no substantial difference in DNA methylation levels when comparing fresh and fresh-frozen tissue samples (Figure S3).

## Discussion

We compared global DNA methylation levels of mouse epidermis obtained with WGBS and BeadChip methods. We also studied the effect of storage conditions using fresh and fresh-frozen mouse epidermis.

We found no substantial difference in DNA methylation levels obtained from fresh and fresh-frozen epidermis analysed with BeadChip and WGBS (Figure 1, Table 1, and Figure 2). DNA methylation levels of fresh and fresh-frozen epidermis from each mouse were strongly correlated using both analysis methods, although the correlation was strongest with the BeadChip method (Figure 2). We recommend either fresh or fresh-frozen epidermis is used for DNA methylation analysis. Using fresh epidermis can give practical limitations as it requires strict time management and planning. This is avoided with fresh-frozen epidermis.

The global DNA methylation levels obtained with WGBS and the BeadChip were strongly correlated (r = 0.984, Figure 3a). This is in accordance with two recent studies reporting high reproducibility between DNA methylation analysis with Illumina’s BeadChip arrays and sequencing-based methods [18,25]. However, these studies involved human tissue samples and used targeted capture sequencing, not WGBS. In our study, the most substantial difference in DNA methylation levels was in the extreme values. The minimum and maximum DNA methylation values obtained with WGBS were 0% and 100% methylation (Table 1). However, with the BeadChip, the minimum and maximum values obtained were 0.35% and 99.56%, respectively (Table 1). Thus, we found that the BeadChip generally overestimated low DNA methylation levels (0–50%) and underestimated high DNA methylation levels (50–100%) when compared with the WGBS results (Figure 4). The same pattern was observed when classifying CpG sites in genomic locations (Figure 6). At CpG islands and CpG island shores where DNA methylation levels were generally low (median < 10%), the BeadChip resulted in higher DNA methylation levels than WGBS. At CpG island shelves and open sea regions with generally higher levels of DNA methylation (median > 50%), the BeadChip resulted in lower methylation levels than WGBS (Figure 6). This was also the main reason for the two distinct clusters in the PCA plot (Figure 3b). The ten CpG sites with the most negative weight, i.e., the ten CpG sites that pulled PC1 towards the left, had WGBS methylation values of 0.00% to 4.35% for all mice. The methylation values of these ten CpG sites obtained with the BeadChip ranged from 5.31% to 25.54% (Table S1). Similarly, the ten CpG sites with the highest positive weight, which pulled PC1 towards the right, showed WGBS methylation values of 84.78% to 100% and BeadChip methylation values of 23.35% to 79.14% (Table S2). It should be noted that using a different normalization method for the analysis of BeadChip data may change the minimum and maximum methylation values, although it is unlikely that the actual measurement of fluorescence will be 0.00% or 100% [24].

We obtained relatively low coverages with WGBS. The average number of reads was 32. Thus, a single read can greatly affect the DNA methylation level. Studies of small DNA methylation differences would require a higher number of reads. For example, at least 100 reads are required to detect a 1% DNA methylation difference. This is a disadvantage of the WGBS method. The BeadChip gives a finer resolution of the methylation levels, but it is unknown how precise the increased resolution is. When we considered the global DNA methylation in 10% methylation intervals, the coverage did not substantially influence the delta values between WGBS and BeadChip DNA methylation levels (Figure 5). Even with coverage below our threshold (< 15 reads), the DNA methylation levels obtained with WGBS and the BeadChip were still strongly correlated (Figure 5b). The results indicated that a lower coverage threshold of, e.g., 10 reads may be acceptable for WGBS studies of DNA methylation levels.

We have only considered global DNA methylation levels due to the limited number of samples in the study. It is important to note that specific CpG sites could differ significantly between fresh and fresh-frozen epidermis and between the BeadChip and WGBS methods. However, for studying the global DNA methylation levels, e.g., for a screening study, we have shown that the WGBS and BeadChip methods perform similarly, and the DNA methylation levels obtained with either method and with either fresh or fresh-frozen epidermis are strongly correlated (Figure 2 and Figure 3).

In conclusion, we showed that DNA methylation analysis of fresh and fresh-frozen epidermis resulted in strongly correlated DNA methylation levels. We also showed that the global DNA methylation levels obtained with WGBS and BeadChip were strongly correlated. There are several things to consider when selecting a method for DNA methylation analysis. For example, WGBS analysis requires only 100 ng DNA, whereas BeadChip analysis requires at least 250 ng per reaction. However, using more DNA for the BeadChip analysis, from 500 ng to 1000 ng, results in higher reproducibility [Illumina; 4]. In addition, much more information is obtained with WGBS compared with BeadChip. This comes at a cost since the price per sample of a WGBS analysis is approximately five times that of a BeadChip analysis. Moreover, the WGBS analysis requires specialized bioinformatics and comprehensive computational power, whereas the BeadChip analysis can be performed with the statistical software R on a desktop computer. Hence, the choice of method ultimately depends on study’s purpose and the availability of equipment and resources.

## Supplementary Material

Supplemental Material

## Data Availability

The data supporting the findings of this study have been deposited in NCBI’s Gene Expression Omnibus (GEO) and are accessible through GEO series accession number GSE213749.
