# Supplementary material for: Comparison of global DNA methylation analysis by whole genome bisulfite sequencing and the Infinium Mouse Methylation BeadChip using fresh and fresh-frozen mouse epidermis
Source: Epigenetics. 2022 Nov 14;18(1):2144574. doi: 10.1080/15592294.2022.2144574 (PMC9980693; doi:10.1080/15592294.2022.2144574)
Supplement: Supplemental Material [file KEPI_A_2144574_SM1282.zip › supplement/Supplementary legend.docx]

# Supplementary data

## Figure S1

**Figure S1:** Report summarising alignment statistics and methylation for 12 samples subjected to WGBS and analysed with Bismark. The file was generated from Bismark.

## Figure S2

**Figure S2:** Plot of the estimated density of methylation levels from fresh and fresh-frozen epidermis from six mice (1-6) analysed with (**A**) the Infinium Mouse Methylation BeadChip (BeadChip) and (**B**) Whole Genome Bisulfite Sequencing (WGBS).

## Figure S3

**Figure S3:** DNA methylation levels of 123,851 CpG sites based on the CpG site location in either CpG Islands (Island), CpG island shores (Shore), CpG island shelves (Shelf), or other genomic locations (Open Sea). The DNA methylation was analysed with BeadChip and WGBS from fresh and fresh-frozen mouse epidermis.

## Table S1

**Table S1:** DNA methylation (%) per sample of the ten CpG sites with the most negative weight in the principal component analysis.

## Table S2

**Table S2:** DNA methylation (%) per sample of the ten CpG sites with the highest positive weight in the principal component analysis.
