# Supplementary material for: Comparison of global DNA methylation analysis by whole genome bisulfite sequencing and the Infinium Mouse Methylation BeadChip using fresh and fresh-frozen mouse epidermis
Source: Epigenetics. 2022 Nov 14;18(1):2144574. doi: 10.1080/15592294.2022.2144574 (PMC9980693; doi:10.1080/15592294.2022.2144574)
Supplement: Supplemental Material [file KEPI_A_2144574_SM1282.zip › supplement/table_s1.docx]

|  | **cg30295371** | **cg46590057** | **cg34477354** | **cg45126195** | **cg39250205** | **cg43185133** | **cg39236477** | **cg43707899** | **cg41623727** | **cg34491992** |
| --- | --- | --- | --- | --- | --- | --- | --- | --- | --- | --- |
| BeadChip | 9.33 | 11.72 | 7.50 | 24.53 | 20.55 | 7.01 | 9.36 | 9.60 | 5.70 | 7.57 |
|  | 9.13 | 12.52 | 7.97 | 25.14 | 22.56 | 6.91 | 9.78 | 9.90 | 6.31 | 8.12 |
|  | 9.70 | 12.35 | 7.82 | 25.54 | 22.38 | 7.54 | 10.73 | 10.28 | 5.87 | 7.62 |
|  | 7.62 | 10.07 | 6.31 | 21.97 | 16.82 | 5.62 | 8.76 | 7.67 | 4.80 | 5.97 |
|  | 8.85 | 12.17 | 7.95 | 24.70 | 22.35 | 7.23 | 9.31 | 10.12 | 5.91 | 7.46 |
|  | 8.85 | 12.17 | 7.95 | 24.70 | 22.35 | 7.23 | 9.31 | 10.12 | 5.91 | 7.46 |
|  | 8.90 | 11.77 | 7.31 | 22.67 | 21.40 | 6.90 | 9.50 | 9.17 | 5.74 | 7.76 |
|  | 9.42 | 12.19 | 8.78 | 25.46 | 22.67 | 7.62 | 10.85 | 10.09 | 6.59 | 7.83 |
|  | 9.48 | 12.90 | 7.66 | 24.97 | 21.53 | 6.85 | 10.09 | 10.28 | 6.35 | 7.96 |
|  | 8.85 | 12.28 | 7.70 | 25.29 | 20.40 | 7.03 | 9.89 | 10.13 | 5.58 | 7.22 |
|  | 8.19 | 11.50 | 6.97 | 22.95 | 19.52 | 6.64 | 8.48 | 9.40 | 5.31 | 6.83 |
|  | 9.26 | 11.99 | 8.13 | 24.94 | 22.02 | 7.57 | 10.26 | 9.94 | 6.21 | 7.91 |
| WGBS | 0.00 | 0.00 | 0.00 | 0.00 | 0.00 | 0.00 | 0.00 | 0.00 | 0.00 | 0.00 |
|  | 0.00 | 0.00 | 0.00 | 0.00 | 0.00 | 0.00 | 0.00 | 0.00 | 0.00 | 0.00 |
|  | 0.00 | 0.00 | 0.00 | 0.00 | 2.56 | 0.00 | 0.00 | 0.00 | 0.00 | 0.00 |
|  | 0.00 | 0.00 | 0.00 | 0.00 | 0.00 | 0.00 | 0.00 | 0.00 | 0.00 | 0.00 |
|  | 0.00 | 0.00 | 0.00 | 0.00 | 0.00 | 0.00 | 0.00 | 0.00 | 0.00 | 0.00 |
|  | 0.00 | 0.00 | 0.00 | 0.00 | 0.00 | 0.00 | 0.00 | 0.00 | 0.00 | 0.00 |
|  | 0.00 | 0.00 | 0.00 | 0.00 | 0.00 | 0.00 | 0.00 | 0.00 | 0.00 | 0.00 |
|  | 0.00 | 0.00 | 0.00 | 0.00 | 4.35 | 0.00 | 0.00 | 0.00 | 0.00 | 0.00 |
|  | 0.00 | 0.00 | 0.00 | 0.00 | 0.00 | 0.00 | 0.00 | 0.00 | 0.00 | 0.00 |
|  | 0.00 | 0.00 | 0.00 | 0.00 | 0.00 | 0.00 | 0.00 | 0.00 | 0.00 | 0.00 |
|  | 0.00 | 0.00 | 0.00 | 0.00 | 2.33 | 0.00 | 0.00 | 0.00 | 0.00 | 0.00 |
|  | 0.00 | 0.00 | 0.00 | 0.00 | 0.00 | 0.00 | 0.00 | 0.00 | 0.00 | 0.00 |

**Table S1:** DNA methylation (%) per sample of the ten CpG sites with the most negative weight in the principal component analysis.
