# Supplementary material for: Comparison of global DNA methylation analysis by whole genome bisulfite sequencing and the Infinium Mouse Methylation BeadChip using fresh and fresh-frozen mouse epidermis
Source: Epigenetics. 2022 Nov 14;18(1):2144574. doi: 10.1080/15592294.2022.2144574 (PMC9980693; doi:10.1080/15592294.2022.2144574)
Supplement: Supplemental Material [file KEPI_A_2144574_SM1282.zip › supplement/table_s2.docx]

|  | **cg41018055** | **cg38723348** | **cg34851202** | **cg38723305** | **cg38723356** | **cg46384028** | **cg30284811** | **cg40124993** | **cg29743545** | **cg39099034** |
| --- | --- | --- | --- | --- | --- | --- | --- | --- | --- | --- |
| BeadChip | 75.12 | 63.54 | 60.97 | 56.13 | 57.96 | 57.70 | 27.29 | 46.26 | 50.85 | 54.56 |
|  | 73.07 | 65.08 | 61.14 | 56.00 | 57.52 | 57.69 | 24.94 | 44.59 | 50.27 | 55.37 |
|  | 74.01 | 64.59 | 59.53 | 56.37 | 57.49 | 57.92 | 23.68 | 45.10 | 52.02 | 54.22 |
|  | 79.14 | 64.47 | 62.15 | 57.28 | 57.90 | 58.42 | 24.34 | 43.73 | 52.73 | 54.80 |
|  | 72.35 | 63.96 | 61.76 | 55.25 | 57.44 | 59.10 | 26.19 | 45.42 | 52.11 | 50.69 |
|  | 72.35 | 63.96 | 61.76 | 55.25 | 57.44 | 59.10 | 26.19 | 45.42 | 52.11 | 50.69 |
|  | 75.12 | 65.24 | 61.15 | 56.24 | 57.12 | 58.59 | 25.50 | 49.43 | 51.52 | 52.09 |
|  | 73.08 | 63.83 | 62.36 | 55.72 | 58.25 | 57.26 | 24.54 | 44.00 | 51.66 | 52.30 |
|  | 73.19 | 63.80 | 60.12 | 55.83 | 58.52 | 59.11 | 24.24 | 44.45 | 50.62 | 53.25 |
|  | 74.45 | 63.95 | 59.79 | 54.54 | 57.14 | 56.84 | 25.60 | 45.59 | 50.26 | 54.59 |
|  | 76.71 | 65.11 | 62.33 | 55.01 | 58.21 | 58.30 | 23.35 | 43.36 | 50.50 | 50.85 |
|  | 74.17 | 63.99 | 62.17 | 55.22 | 57.17 | 58.14 | 25.38 | 44.45 | 51.63 | 50.29 |
| WGBS | 100.00 | 92.36 | 100.00 | 88.73 | 88.24 | 85.16 | 100.00 | 97.62 | 95.24 | 100.00 |
|  | 100.00 | 91.63 | 100.00 | 88.08 | 88.03 | 86.47 | 97.14 | 95.00 | 97.14 | 100.00 |
|  | 98.70 | 92.33 | 100.00 | 88.46 | 88.10 | 86.61 | 100.00 | 94.55 | 91.49 | 97.73 |
|  | 95.00 | 91.41 | 95.00 | 87.53 | 88.33 | 85.89 | 95.65 | 94.12 | 94.74 | 100.00 |
|  | 100.00 | 91.87 | 100.00 | 86.62 | 88.06 | 85.35 | 100.00 | 97.14 | 100.00 | 98.04 |
|  | 97.22 | 92.40 | 100.00 | 87.40 | 88.24 | 85.22 | 100.00 | 100.00 | 96.30 | 100.00 |
|  | 100.00 | 91.87 | 100.00 | 87.50 | 87.96 | 85.35 | 100.00 | 100.00 | 100.00 | 100.00 |
|  | 100.00 | 92.21 | 95.00 | 88.51 | 88.44 | 86.28 | 100.00 | 94.12 | 100.00 | 100.00 |
|  | 96.77 | 91.94 | 100.00 | 88.36 | 88.30 | 86.17 | 100.00 | 95.65 | 100.00 | 100.00 |
|  | 100.00 | 92.02 | 100.00 | 87.73 | 88.34 | 86.73 | 97.22 | 95.45 | 92.11 | 97.30 |
|  | 97.37 | 92.14 | 100.00 | 86.62 | 88.38 | 84.78 | 100.00 | 97.06 | 94.12 | 95.24 |
|  | 100.00 | 92.79 | 100.00 | 88.77 | 88.43 | 86.97 | 100.00 | 96.55 | 100.00 | 100.00 |

**Table S2:** DNA methylation (%) per sample of the ten CpG sites with the highest positive weight in the principal component analysis.
