# Supplementary figures and images for: Comparison of global DNA methylation analysis by whole genome bisulfite sequencing and the Infinium Mouse Methylation BeadChip using fresh and fresh-frozen mouse epidermis
Source: Epigenetics. 2022 Nov 14;18(1):2144574. doi: 10.1080/15592294.2022.2144574 (PMC9980693; doi:10.1080/15592294.2022.2144574)

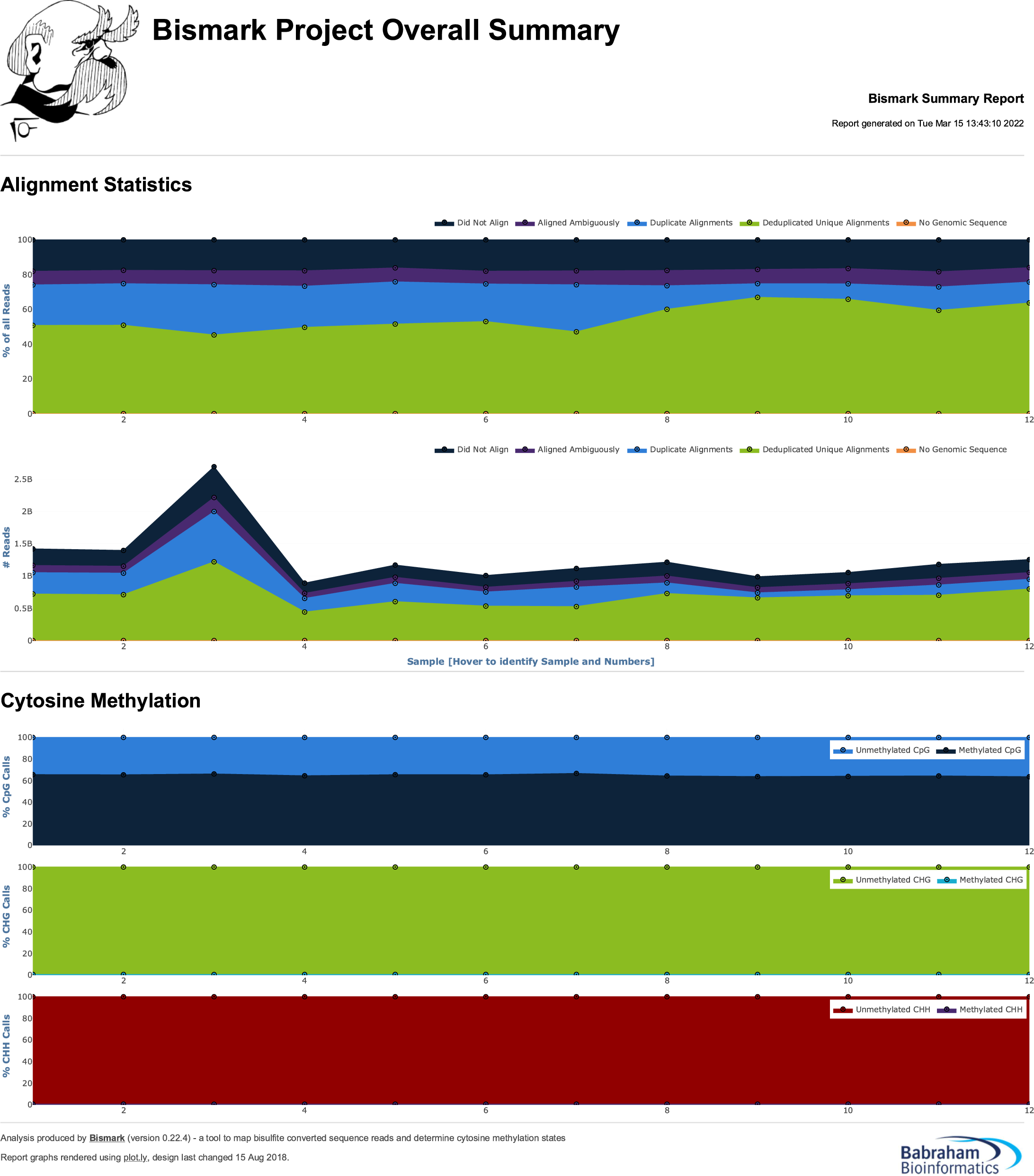

Supplement: Supplemental Material [file KEPI_A_2144574_SM1282.zip › supplement/fig_s1.tif]
